# Male Germ Cell Specification in Plants

**DOI:** 10.3390/ijms25126643

**Published:** 2024-06-17

**Authors:** Wenqian Chen, Pan Wang, Chan Liu, Yuting Han, Feng Zhao

**Affiliations:** 1 Shaanxi Key Laboratory of Qinling Ecological Intelligent Monitoring and Protection, School of Ecology and Environment, Northwestern Polytechnical University, Xi’an 710129, China; 2023070027@nwpu.edu.cn (W.C.); wangpan2023@mail.nwpu.edu.cn (P.W.); chanliu@nwpu.edu.cn (C.L.); hanyt@mail.nwpu.edu.cn (Y.H.); 2 Collaborative Innovation Center of Northwestern Polytechnical University, Shanghai 201108, China

**Keywords:** germ cell, meristem, anther, morphogenesis

## Abstract

Germ cells (GCs) serve as indispensable carriers in both animals and plants, ensuring genetic continuity across generations. While it is generally acknowledged that the timing of germline segregation differs significantly between animals and plants, ongoing debates persist as new evidence continues to emerge. In this review, we delve into studies focusing on male germ cell specifications in plants, and we summarize the core gene regulatory circuits in germ cell specification, which show remarkable parallels to those governing meristem homeostasis. The similarity in germline establishment between animals and plants is also discussed.

## 1. Introduction

Generally, multicellular organisms initiate their life cycle from a single totipotent cell. As cell division progresses and ordered multicellular organization ensues, certain cells are specified as germ cells (Table 1, glossary), responsible for transmitting genetic information from one generation to the next. In most higher animals, the germline (Table 1) is segregated early during embryogenesis [1]. Primordial germ cells (PGCs), distinguished from somatic cells (Table 1), serve as bipotential precursors of eggs and sperms, developing into eggs in the ovary and sperm in the testes [1,2]. Unlike animals, the architecture of higher plants primarily arises from post-embryonic development. In plants, it is generally understood that the germline is segregated late during development post-embryonically and tightly coupled with organogenesis. In comparison to animals, the embryogenesis of plants is relatively rudimentary. During embryogenesis, the basic apical–basal axis is established, characterized by the formation of the shoot apical meristem (SAM) and root apical meristem (RAM) at opposite poles of the embryo. Subsequently, during post-embryonic development, the SAM and RAM give rise to the entire aerial and underground structures, respectively.

**Figure 1:**
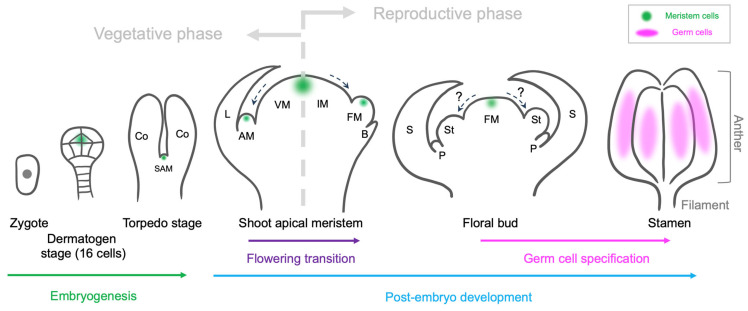
A schematic representation of the male germ cell specification route in the model plant Arabidopsis. Dashed arrows indicate the ‘detachment’ of meristem cells. Co, cotyledon; SAM, shoot apical meristem; VM, vegetative meristem; AM, axillary meristem; L, leaf; IM, inflorescence meristem; FM, floral meristem; B, bract; St, stamen; P, petal; S, sepal.

In a representative dicot, Arabidopsis, SAM precursor cells are initially distinguishable in dermatogen-stage (16-cell) embryos [3] (Figure 1). During the vegetative development phase, the SAM generates leaves at its periphery. At the boundary between the SAM and leaf primordia, a group of cells are ‘detached’ from the SAM and sequestered earlier from the surroundings, undergoing minimal cell division [4] (Figure 1). These initial axillary meristem (AM) precursors express *SHOOT MERISTEMLESS* (*STM*) and, through the activation of *WUSCHEL*(*WUS*) and *CLAVATA 3* (*CLV3*), give rise to new functional SAMs, thereby generating new branches [5,6]. This process recurs throughout the plant body, contributing to the formation of a complex architecture [7]. During the reproductive phase, the developmental identity of the SAM transitions to the inflorescence meristem (IM), accompanied by changes in its doming shape [8] and gene regulatory network (GRN) [9] (Figure 1). In Arabidopsis, similar to the process of AM formation, the floral meristem (FM) initiates at the axis of cryptic bract primordia (Figure 1). The key factors in meristem maintenance, namely, *WUS* and *CLV3*, are reactivated at the center of the FM to sustain meristematic activity, leading to the initiation of floral organs in four concentric whorls (from outer to inner: sepals, petals, stamens, and carpels) (Figure 1). Male and female germ cells are progressively specified within specific inner tissues of the stamens and carpels, indicating the close link between germ cell specification and the morphogenesis of reproductive organs. In this paper, we will review our current understanding of male germ cell specification in plants, primarily using Arabidopsis as an example.

## 2. Stamen Morphogenesis

### 2.1. Pattern Formation: Spatial–Temporal Initiation of Stamen Primordia

**Figure 2:**
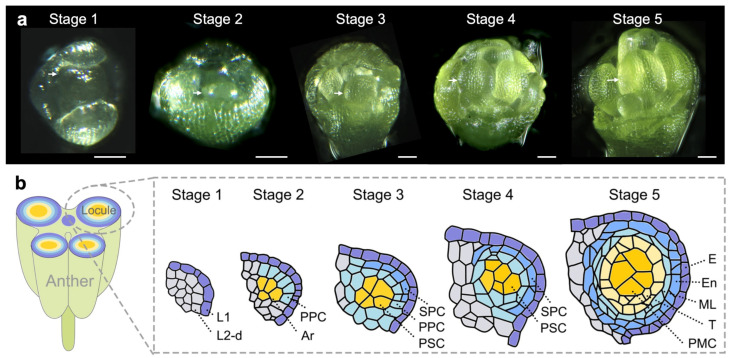
Anther development in the model plant Arabidopsis. (**a**) The successive developmental stages of stamens in Arabidopsis flowers. White arrow heads indicate the anthers at the distal part of stamen primordia. Scale bars, 50 µm. (**b**) A schematic representation of the germ cell specification process in Arabidopsis anthers. (Left) A cross-section of the anther. (Right) The evolution of germ cell specification in an anther lobe at different stages. Cell types are marked out with different colors. L1, the outermost cell layer; L2-d, cells derived from the second layer; PPC, primary parietal cell; Ar, archesporial cell; SPC, secondary parietal cell; PSC, primary sporogenous cell; E, epidermis; En, endothecium; ML, middle layer; T, tapetum. Images are taken from PNAS [25] with permission.

Different from animals, plant cells cannot move, and thus, germ cell specification must be tightly coordinated with organogenesis. In flowering plants, male germ cells are embedded in the male reproductive organ—the stamen. The number of stamens varies among species, from a few to dozens. In Arabidopsis, there are six stamens containing a distal structure, called the anther (where germ cells are located), and a proximal structure, called the filament (responsible for supporting and transporting nutrients). In the wild-type floral bud, four medial (long) stamen primordia arise initially along the periphery of the FM, with two lateral (short) stamen primordia initiating later [10] (Figure 2a). The underlying mechanism governing the synchronization of the timing and positional development of stamen primordia remains unclear.

All lateral organs originate from the shoot apical meristem or its derivates (i.e., axillary meristem and floral meristem). Physical (e.g., size, shape, mechanical properties) and biochemical factors (e.g., phytohormones) are widely incorporated in the regulation of the number and spatial–temporal distribution of leaves and floral buds at the shoot apical meristem [11,12,13,14,15,16]. Several pieces of evidence suggest that a similar mechanism may also apply to floral organogenesis. Indeed, dynamic auxin distribution and auxin biosynthesis are both crucial for regulating floral organ number and identity [17,18]. In addition, a recent study by Zhu and colleagues revealed that the timing of sepal initiation is regulated by *DEVELOPMENT RELATED MYB-LIKE 1* (*DRMY1*) via focusing the auxin and cytokinin signaling pattern [19]. This mechanism is very similar to the robustness of the phyllotactic pattern control at the SAM, underscoring the importance of two distinct hormone-based fields for achieving robust pattern formation [14,20]. 

Meristem size and activity play significant roles in organ number control. For instance, *WIGGUM* (*WIG*) functions in parallel with other genes like *CLAVATA* (*CLV*) to regulate meristem structure and floral organ number [21]. Conversely, mutations in *PERIANTHIA* (*PAN*) result in increased sepal and petal numbers but decreased stamen numbers, independent of the floral meristem size and floral identity genes [22]. Another boundary gene, *SUPERMAN* (*SUP*), expressed at the boundary between whorl 3 and whorl 4, is responsible for floral meristem termination and the repression of stamen primordia’s initiation from whorl 4 [23] through fine-tuning local auxin biosynthesis via interaction with polycomb repressive complex 2 (PRC2) [24]. Although auxin is incorporated in the process of stamen initiation, whether stamen primordia follow a specific phyllotactic pattern and whether physical factors participate in regulating stamen initiation remain largely unexplored.

### 2.2. Stamen Polarity: Establishment of Three AXES

Once bulging out from the meristem, the stamen primordia quickly adopt a three-dimensional structure with three axes: adaxial–abaxial, medial–lateral, and proximal–distal (Figure 2a). Similar to other lateral organs, stamens are believed to originate from leaves [26]. 

**Adaxial–abaxial axis**. In Arabidopsis, leaf primordia initiate at the flank of the SAM with a slightly ellipsoid shape. However, leaf polarity is determined much earlier than the initial outgrowth by a group of factors such as auxin, small RNAs, HD-ZIP III family proteins, *ASYMMETRIC LEAVES 2* (*AS2*), and *KANADI* (*KAN*) genes. These factors interact and sequentially define the adaxial–abaxial polarities (also called dorsoventrality) and the boundaries in between [27,28,29,30,31,32,33]. Once leaf primordia bulge out, a microtubule-mediated mechanical feedback mechanism is proposed to sustain or even amplify the leaf flatness [34]. 

In contrast to leaves, stamen primordia initiate in the inner third whorl of the floral meristem (FM) with approximately 28 cells in the initium, based on live imaging observation [35]. The shape of anther primordia is initially isotropic, resembling a radially symmetric disc from a cross-section view [36,37]. Around four days after initiation, the distal part of stamen primordia begins to grow more isotropically, corresponding to the formation of the anther (where germ cells will reside in) [35]. Subsequently, from a transverse section view, the shape of the anther shifts from a disk to a trapezoid (from around anther stage 2) and finally to a butterfly shape by anther stage 5 [36,37]. This symmetry transition is documented in rice anthers, where the polarity genes *Oryza sativa ETTIN1* (*OsETT1*) (*Auxin Response Factor*, *ARF* gene) and *PHB3* (*DH-ZIP III* family gene) undergo a shift in gene expression. *OsETT1* is initially expressed on the abaxial side of anthers, and later on, it is located at both the adaxial and abaxial sides. By contrast, *PHB3* expression shifts from the adaxial side to the intermediate domain of the adaxial and abaxial anther lobes [38]. Similarly, symmetry transition in *Canna indica* anthers is reported to be correlated with the rearrangement of *CiPHB* (adaxial gene) and *CiFIL* (abaxial gene) [39]. In Arabidopsis flowers, *PHB* expression initially occurs on the adaxial side but later becomes restricted to the two lateral domains of anther primordia [40], which is very similar to what has been found in rice anthers [38]. By contrast, *ETTIN* (*ETT*) is initially expressed in the abaxial domain of anther primordia; however, at least around stages 4 and 5, *ETT* is abundant at the procambial strand and inner borders of locules [41]. This expression pattern is very different from the *FIL* expression pattern, which, along with other *YABBY* genes (*YABBY2* and *YABBY3*), is localized abaxially in anther primordia until at least the butterfly shape of the anther is formed [42]. While the genetic manipulation of adaxial–abaxial gene expression in leaves can generate four-bladed leaves, mimicking rice anthers [29], the dynamics of dorsoventrality in Arabidopsis anthers require further elucidation.

**Medial–lateral axis**. Another aspect of three-dimensional organ shape is along the medial–lateral direction. During leaf development, medial–lateral polarity is mediated by two *WUSCHEL-RELATED HOMEOBOX* (*WOX*) family genes, *WOX1* and *PRESSED FLOWER* (*PRS*). These genes are conserved in several flowering plants and play a role in controlling leaf and many lateral organ expansions [28,29,43,44,45,46]. While *PRS* is involved in sepal development [28,47], the petals and stamens are fairly normal in *prs* [47].

**Proximal–distal axis**. Proximal–distal polarity is established as the stamen primordia grow away from the floral meristem, resulting in a distal anther with bilateral symmetry and a proximal filament with radial symmetry. However, in some basal taxa, the filaments maintain the adaxial–abaxial polarity, exhibiting a flat structure [48]. The distinct polarity control along the proximal–distal axis is still elusive. In leaf development, BLADE-ONPETIOLE 1 and 2 (BOP1/2) are well-known factors controlling the specification of the leaf petiole domain. In *bop1 bop2* mutants, the petiole domain is diminished and is overtaken by an extra blade, whereas the anther and filament development remain largely unaffected [49]. In Arabidopsis stamens, *NUBBIN* (*NUB*) and *JAGGED* (*JAG*) redundantly regulate stamen proximal–distal polarity. In *nub jag* mutants, distal anther development is severely disrupted, sometimes resulting in an abaxialized radial symmetric distal structure [40]. In fact, while the shifting of dorsoventrality is necessary for the establishment of anther shape in rice, it is assumed that the adaxial polarity might be lost in the proximal part of the stamen, leading to an abaxialized filament [38]. The fact that anthers exhibit a radialized shape in certain polarity mutants, such as *kan*, *phb-1d*, and *fil* [38,50,51,52,53], indicates that the adaxial–abaxial polarity might also play a role in coordinating proximal–distal axial specification.

## 3. Germ Cell Specification: Anther Growth and Cellular Differentiation

### 3.1. Growth Dynamics of Anthers

The most prominent function of the anther is to produce male germ cells. Based on the anther shape and size, the staging for early anther development has been elaborately described [36,37,54,55]. Recently, taking advantage of live imaging and quantitative image analyses, Silveira and colleagues provided detailed insights into stamen morphodynamics in Arabidopsis [35]. Along the proximal–distal axis, rapid growth initially occurs at the tip of anther primordia, corresponding to the differentiation of the anther and filament. Subsequently, growth ceases, also starting from the anther tip. This basipetal differentiation pattern is reminiscent of what Gould and Lord proposed in lily anther: there is a growth wave from the tip to the base that could be correlated with auxin flow [56], although such a growth wave is not observed in maize anthers [57]. Another difference in the anther growth pattern between Arabidopsis and maize is along the adaxial–abaxial axis. While the development of anther lobes is highly canalized and synchronized in maize [58], it is proposed to be temporally different in Arabidopsis, possibly due to variations in the growth rates between the adaxial and abaxial lobes [35]. Along the medial–lateral axis, the growth rate is higher in the lateral domain. This growth pattern is fascinating, suggesting that heterogeneous growth might be relevant to the internal tissue growth of the anther lobe [35]. 

### 3.2. Concentric Layered Structure and GC Formation

It has been widely accepted that male germ cells are initiated from L2 cells in the stamen primordia, whereas their traceability back to the shoot apical meristem remains hypothetical [55,58,59,60,61]. The initial form of a germ cell in the anther, known as the archesporial cell (AR, Table 1), is characterized by its large size and nucleus [25,37,55]. After several rounds of mitosis, these ARs become mature to form so-called pollen mother cells (PMCs) or microsporocytes, which ultimately undergo meiosis. As the germ cell lineage (Table 1) is specified, surrounding somatic cells divide in an orderly manner and form a concentric layered structure. In each somatic layer, the cells adopt a unique cell fate, including the tapetum, middle layer, endothecium, and epidermis from the innermost to the outermost layer (Figure 2b). The number of each type of somatic cell layer may vary among different species [48].

Two different models are proposed to explain the formation of these concentric multilayer structures. In the lineage model, ARs localized at L2 are proposed to divide asymmetrically, with the smaller daughter cell generating the outer somatic layers and interior larger daughter cells serving as germinal lineage founders. In contrast, the positional model suggests that there is no clear enlargement of hypodermal ARs and no subsequent asymmetric division in maize anthers. Instead, the ARs are proposed to initiate from the innermost of a small group of L2-derived cells with a population of around 100 [57]. While these differences may probably be species-dependent, among all the somatic cell types, the tapetum appears to be present and conserved at least across flowering plants, with the function of nursing the germ cells [48,61]. By contrast, the function of the middle layer remains unclear [61,62,63], and its presence varies among species [48]. Advances in imaging techniques, single-cell sequencing, and spatial transcriptomics are expected to help us find more factors responsible for the specification of different cell types, as well as their interactions and functionalities (e.g., [62,64,65]).

## 4. Germ Cell Specification: Molecular Players

**Figure 3:**
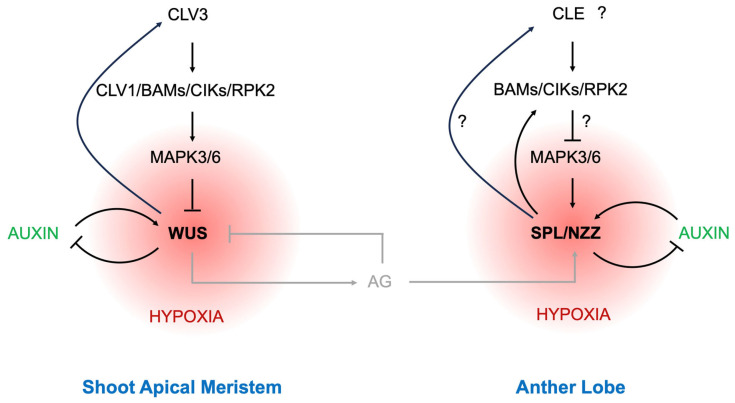
Gene regulatory circuits that regulate meristem cell homeostasis and germ cell specification. Red circles indicate the hypoxia gradients.

In the past few decades, plenty of factors involved in regulating germ cell fate determination have been identified (for comprehensive reviews, see [37,55,61,63,66,67,68]). In the gene regulatory network (GRN), *SPROCYTLESS/NOZZLE* (*SPL/NZZ*) emerges as a central hub. *SPL/NZZ* was first identified in Arabidopsis, playing a pivotal role in specifying both male and female germ cells [69,70], and its homologs have been recently identified in tomato, rice, and cucumber [71,72,73,74]. Interestingly, while it is suggested that there is no meristem in anthers [55], the *SPL/NZZ*-centered circuit in germ cell specification bears striking similarities to the WUS-centered circuit in regulating shoot apical meristem activity (Figure 3). In these circuits, a group of factors like leucine-rich repeat receptor-like protein kinases (LRR-RLKs), mitogen-activated protein kinases, hypoxia, and auxin are involved in forming complex feedback loops (Figure 3). In this section, we will briefly compare the similarities between the regulation of SAM homeostasis and the specification of GC-specific processes.

LRR-RLKs are key factors participating in a wide range of developmental processes. Among them, BARELY ANY MERISTEM 1/2/3 (BAM1/2/3), CLAVATA3 INSENSITIVE RECEPTOR KINASES (CIKs), and RECEPTOR-LIKE PROTEIN KINASE 2 (RPK2) are well known in regulating germ cell specification and meristem activity [75,76,77,78]. Plant RLKs share structural similarities with animal receptor tyrosine kinase (RTK) and exhibit similar downstream components, such as the MAPK cascade and ROS production [79]. In the shoot apical meristem, MPK3/6 are activated by CLV3 signals through RLKs (mainly CLV1 and BAM1) to regulate meristem homeostasis by repressing WUS expression [80], while MPK3/6 can stabilize SPL/NZZ by direct phosphorylation to ensure germ cell specification [81].

In animals, the maintenance of the pluripotent state of stem cells requires hypoxic conditions, whereas higher oxygen tension promotes cell differentiation [82]. In the plant SAM, there is an increase in the oxygen gradient from the inner to outer layers. The inhibition of the hypoxic response by exposing plantlets to hyperoxic conditions impairs meristem activity [83]. Interestingly, the redox status has also been implicated as a determinant for germ cell specification in maize anthers [84]. The hypoxia status in maize anthers is regulated by the glutaredoxin gene *MALE STERILE CONVERTED ANTHER 1* (*MSCA1*) [84]. While the redox status in other plant anthers has not been experimentally measured, the mutation of *MSCA1* homolog genes in Arabidopsis and rice also leads to defects in germ cell formation [85,86], suggesting a plausible conservative function of hypoxia in germ cell specification at least among angiosperms.

It has long been noticed that auxin and its related signaling components are present at the summit and inner core of the SAM [87,88]. Recent evidence indicates that auxin biosynthesis and transport are essential for *WUS* expression, and WUS, in turn, rheostatically controls the auxin signaling pathway via epigenetic regulations [89,90]. Similar to this WUS–auxin feedback, SPL/NZZ–auxin feedback has recently been identified in Arabidopsis anthers, where *TRYPTOPHAN AMINOTRANSFERASE OF ARABIDOPSIS 1* (*TAA1*)/*TRYPTOPHAN AMINOTRANSFERASE RELATED 2* (*TAR2*)-mediated auxin biosynthesis is crucial for the centripetal auxin distribution pattern and GC specification by the activation of *SPL/NZZ* expression. SPL/NZZ, in turn, mediates the effect of auxin on GC specification and modifies auxin biosynthesis to maintain a centripetal auxin distribution [25]. Apart from auxin, brassinosteroid (BR) signaling has also been implicated in regulating *SPL/NZZ* expression and thus GC formation [91,92,93]. However, while BRs are important for organ boundary formation in the SAM, their functions in the central meristem remain unclear [94]. 

Despite the intriguing parallels between SAM homeostasis and germ cell-specific regulation, there exists a physical separation of the SAM and anther lobes in space and time. In Arabidopsis, the homeotic gene *AGAMOUS* (*AG*) plays a crucial role in bridging the gap (Figure 3). As a bifunctional transcription factor, WUS acts as a repressor in regulating the cell stemness in the SAM and as an activator in regulating floral patterning via inducing *AG* expression [95]. Once activated in the floral meristem (FM), AG in turn represses WUS by activating *KNUCKLES* (*KNU*) transcription [96,97,98,99]. In *ag* mutants, all floral organs are transformed into sepals and petals, with no germ cell formation. The ectopic expression of *AG* in *ag* directly activates *SPL/NZZ* transcription, hence inducing germ cell formation in petals [100]. Moreover, with the accumulation of AG, the petals in *ag* flowers can ultimately be transformed into stamens [100,101]. This indicates that AG is also a factor bridging anther morphogenesis and germ cell specification. 

In fact, accumulating evidence is beginning to reveal how anther formation may be linked to its function as a germ cell container at the molecular level. For example, recent work by Zheng and colleagues demonstrated that auxin, a morphogen (Table 1) in plants, acts as a key factor in guiding male germ cell specification in Arabidopsis anthers [25]. This echoes the involvement of many factors that regulate organ polarity, which is also auxin-related, that also mediate germ cell formation by interacting with SPL/NZZ (e.g., YAABY, ETTIN) [102,103].

## 5. Discussion and Perspectives

Understanding how germ cells are originally specified is a captivating question in developmental biology. It has been a long-lasting notion that plants and animals employ different strategies to establish their germlines. However, recent research suggests that this may not always be the case. For instance, in the majority of animal classes, the germline segregates after embryogenesis, a process very similar to what occurs in plants [104]. By contrast, in vertebrates and ecdysozoans, germline segregation, as commonly observed, occurs during embryogenesis [104]. One explanation for these differences might be motility: if an organism is more motile, like vertebrates and ecdysozoans, it is much easier to shield the germline from fluctuating environments. Conversely, if the organism is motile-limited or even sessile, like some lower animals and plants, germline segregation may be reasonably delayed or more tunable to buffer against environmental fluctuations. For example, cnidarians (corals and jellyfish) possess high regenerative ability and do not succumb to age. These regenerative abilities and plant-like clonal growth rely on a group of adult stem cells called i-cells, which are proven to have the capability to differentiate into all somatic lineages and germ cells [105].

In flowering plants, much like animal stem cells, groups of meristematic cells are found in the shoot apex, root apex, and also the stem [106]. Within the shoot apical meristem, a CLV-WUS feedback loop maintains meristem homeostasis, balancing cell self-renewal and differentiation to orchestrate all areal organ formation (including determinate lateral organs and indeterminate new branches). Interestingly, while anthers are not typically considered to possess a meristem [55], a SPL/NZZ-based circuit that determines the germ cell specification in Arabidopsis anther lobes closely resembles the CLV-WUS-based circuit structure regulating SAM activity (Figure 3). This suggests there might be a very tight link between meristematic cells and germ cells in plants. This notion is reinforced by recent research indicating that two ARGONAUTE (AGO) proteins (AGO5 and AGO9) mark both meristematic cells in the SAM and GCs in reproductive organs, implying a protective function of AGOs in safeguarding the genome from damage by transposons [107]. Thus, while plants and animals diverged early in evolutionary history [108], they may have independently evolved similar mechanisms to preserve germ cell potential in meristematic or stem cells, where the mutation rate in the genome is relatively low [4,107,109]. 

While this comparison is intriguing, several questions remain. For instance, evolutionarily, the function of WUS appears later than that of CLVs. Thus, CLV–cytokinin cross-talk and its mediated auxin homeostasis have recently been proposed to be a more ancient property for SAM homeostasis [110,111,112]. Additionally, the homologs of SPL/NZZ have only been identified in angiosperms. It remains unknown whether SPL/NZZ-like proteins exist in other plant species, particularly early land plants like bryophytes. Moreover, auxin has been proven to be essential for the development of meristem function in Marchantia [113,114]. However, the function of auxin in germ cell specification in bryophytes is not clear. What could be the simplest circuit conserved evolutionarily for germ cell induction and probably also for meristematic cell fate acquisition is an open question. 

Another interesting factor that commonly guides germ cell specification in both animals and plants is morphogen (Table 1). In animals, retinoic acid (RA) exhibits properties characteristic of morphogen by providing positional information to cells and directing their differentiation and patterning during animal development. RA concentration gradients play a critical role in coordinating complex developmental processes and ensuring the proper formation of tissues and organs in various animal species, which is very comparable to the function of auxin in plant development [115,116]. Notably, during the development of the reproductive system, retinoic acid is essential for the formation of gonads and germ cells in various animal models [117,118,119]. This parallels the role of auxin in germ cell specification in Arabidopsis anthers [25]. 

The last but not least interesting question is the coordination of germ cell specification in plants. In Brassicaceae, for instance, the proportion of flowers (harboring germ cells) initiated on secondary inflorescences (transformed from the axillary meristem) represents around half of the total floral production, and secondary inflorescences continue to mutually inhibit each other. Walker and Bennett hypothesized that this phenomenon can be explained by the extension of the canalization model for dominance and/or shoot branching [120], which is known to be regulated by auxin transport. Additionally, this coordination might be associated with the depletion of meristematic potential at the shoot apical meristem. Since developmental timing in plants is proposed to be strongly dependent on growth [121], auxin, as a key factor of both long-distance and local signals in orchestrating plant growth and morphogenesis, might be a good candidate for further investigation into its potential role in coordinating germ cell specification throughout the plant. With the rapid advancement of cutting-edge imaging techniques, computational modeling, and the emergence of novel model systems (e.g., [122]), we are poised to gain a deeper understanding of how plants specify their germ cells.

## Figures and Tables

**Table 1:** Glossary.

**Germ cell**: A germ cell, also known as a gamete precursor cell or reproductive cell, typically arises from PGCs during early embryonic development in animals. Germ cells undergo meiosis to form gametes (sperm or eggs) in animals and plants, delivering genetic information from one generation to their offspring.
**Germline:** A line of cells that separate from somatic cells and become germ cells.
**Primordial germ cell**: A primordial germ cell (PGC) is a specialized cell type that gives rise to gametes (sperm and eggs) in animals during embryonic development. These cells are set aside early in embryonic development and undergo a unique series of developmental events that ultimately lead to their differentiation into mature gametes.
**Somatic cells**: Cells in the organism that are not germ cells.
**Archesporial cell (AR cell)**: These cells give rise to the spore mother cell, which will further undergo meiosis to produce spores in plants. AR cells are specified early within the floral meristem or sporophytic tissue, which could be analogous to animal PGCs.
**Germ cell lineage**: The cells that give rise to the gametes, including the primordial germ cells, the developing eggs and sperms, and the mature gametes.
**Morphogen**: A morphogen is a signaling molecule that diffuses through tissues and forms a concentration gradient, providing positional information to cells and directing their differentiation and patterning during development.

## References

[1] Hansen C.L., Pelegri F. (2021). Primordial Germ Cell Specification in Vertebrate Embryos: Phylogenetic Distribution and Conserved Molecular Features of Preformation and Induction. Front. Cell Dev. Biol..

[2] Kanamori M., Oikawa K., Tanemura K., Hara K. (2019). Mammalian Germ Cell Migration during Development, Growth, and Homeostasis. Reprod. Med. Biol..

[3] Dresselhaus T., Jürgens G. (2021). Comparative Embryogenesis in Angiosperms: Activation and Patterning of Embryonic Cell Lineages. Annu. Rev. Plant Biol..

[4] Burian A., Barbier de Reuille P., Kuhlemeier C. (2016). Patterns of Stem Cell Divisions Contribute to Plant Longevity. Curr. Biol..

[5] Shi B., Zhang C., Tian C., Wang J., Wang Q., Xu T., Xu Y., Ohno C., Sablowski R., Heisler M.G. (2016). Two-Step Regulation of a Meristematic Cell Population Acting in Shoot Branching in Arabidopsis. PLoS Genet..

[6] Yang T., Jiao Y., Wang Y. (2022). Stem Cell Basis of Shoot Branching. Plant Cell Physiol..

[7] Bai S.-N. (2019). Plant Morphogenesis 123: A Renaissance in Modern Botany?. Sci. China Life Sci..

[8] Kinoshita A., Vayssières A., Richter R., Sang Q., Roggen A., van Driel A.D., Smith R.S., Coupland G. (2020). Regulation of Shoot Meristem Shape by Photoperiodic Signaling and Phytohormones during Floral Induction of Arabidopsis. eLife.

[9] Quiroz S., Yustis J.C., Chávez-Hernández E.C., Martínez T., de la Sanchez M.P., Garay-Arroyo A., Álvarez-Buylla E.R., García-Ponce B. (2021). Beyond the Genetic Pathways, Flowering Regulation Complexity in Arabidopsis thaliana. Int. J. Mol. Sci..

[10] Smyth D.R., Bowman J.L., Meyerowitz E.M. (1990). Early Flower Development in Arabidopsis. Plant Cell.

[11] Landrein B., Refahi Y., Besnard F., Hervieux N., Mirabet V., Boudaoud A., Vernoux T., Hamant O. (2015). Meristem Size Contributes to the Robustness of Phyllotaxis in Arabidopsis. J. Exp. Bot..

[12] Zhao F., Chen W., Traas J. (2018). Mechanical Signaling in Plant Morphogenesis. Curr. Opin. Genet Dev..

[13] Zhao F., Chen W., Sechet J., Martin M., Bovio S., Lionnet C., Long Y., Battu V., Mouille G., Monéger F. (2019). Xyloglucans and Microtubules Synergistically Maintain Meristem Geometry and Phyllotaxis. Plant Physiol..

[14] Vernoux T., Besnard F., Godin C. (2021). What Shoots Can Teach about Theories of Plant Form. Nat. Plants.

[15] Trinh D.-C., Alonso-Serra J., Asaoka M., Colin L., Cortes M., Malivert A., Takatani S., Zhao F., Traas J., Trehin C. (2021). How Mechanical Forces Shape Plant Organs. Curr. Biol..

[16] Zhao F., Long Y. (2022). Mechanosensing, from Forces to Structures. Front. Plant Sci..

[17] Benková E., Michniewicz M., Sauer M., Teichmann T., Seifertová D., Jürgens G., Friml J. (2003). Local, Efflux-Dependent Auxin Gradients as a Common Module for Plant Organ Formation. Cell.

[18] Cheng Y., Dai X., Zhao Y. (2006). Auxin Biosynthesis by the YUCCA Flavin Monooxygenases Controls the Formation of Floral Organs and Vascular Tissues in Arabidopsis. Genes Dev..

[19] Zhu M., Chen W., Mirabet V., Hong L., Bovio S., Strauss S., Schwarz E.M., Tsugawa S., Wang Z., Smith R.S. (2020). Robust Organ Size Requires Robust Timing of Initiation Orchestrated by Focused Auxin and Cytokinin Signalling. Nat. Plants.

[20] Besnard F., Refahi Y., Morin V., Marteaux B., Brunoud G., Chambrier P., Rozier F., Mirabet V., Legrand J., Lainé S. (2013). Cytokinin Signalling Inhibitory Fields Provide Robustness to Phyllotaxis. Nature.

[21] Running M.P., Fletcher J.C., Meyerowitz E.M. (1998). The WIGGUM Gene Is Required for Proper Regulation of Floral Meristem Size in Arabidopsis. Development.

[22] Running M.P., Meyerowitz E.M. (1996). Mutations in the PERIANTHIA Gene of Arabidopsis Specifically Alter Floral Organ Number and Initiation Pattern. Development.

[23] Prunet N., Yang W., Das P., Meyerowitz E.M., Jack T.P. (2017). *SUPERMAN* Prevents Class B Gene Expression and Promotes Stem Cell Termination in the Fourth Whorl of *Arabidopsis thaliana* Flowers. Proc. Natl. Acad. Sci. USA.

[24] Xu Y., Prunet N., Gan E.-S., Wang Y., Stewart D., Wellmer F., Huang J., Yamaguchi N., Tatsumi Y., Kojima M. (2018). SUPERMAN Regulates Floral Whorl Boundaries through Control of Auxin Biosynthesis. EMBO J..

[25] Zheng Y., Wang D., Ye S., Chen W., Li G., Xu Z., Bai S., Zhao F. (2021). Auxin Guides Germ-Cell Specification in Arabidopsis Anthers. Proc. Natl. Acad. Sci. USA.

[26] Von Goethe J.W. (1790). Versuch Die Metamorphose Der Pflanzen Zu Erklären.

[27] Wang Q., Marconi M., Guan C., Wabnik K., Jiao Y. (2022). Polar Auxin Transport Modulates Early Leaf Flattening. Proc. Natl. Acad. Sci. USA.

[28] Zhao F., Traas J. (2021). Stable Establishment of Organ Polarity Occurs Several Plastochrons before Primordium Outgrowth in Arabidopsis. Development.

[29] Caggiano M.P., Yu X., Bhatia N., Larsson A., Ram H., Ohno C.K., Sappl P., Meyerowitz E.M., Jönsson H., Heisler M.G. (2017). Cell Type Boundaries Organize Plant Development. eLife.

[30] Scacchi E., Paszkiewicz G., Thi Nguyen K., Meda S., Burian A., De Back W., Timmermans M.C.P. (2024). A Diffusible Small-RNA-Based Turing System Dynamically Coordinates Organ Polarity. Nat. Plants.

[31] Burian A., Paszkiewicz G., Nguyen K.T., Meda S., Raczyńska-Szajgin M., Timmermans M.C.P. (2022). Specification of Leaf Dorsiventrality via a Prepatterned Binary Readout of a Uniform Auxin Input. Nat. Plants.

[32] Qi J., Wang Y., Yu T., Cunha A., Wu B., Vernoux T., Meyerowitz E., Jiao Y. (2014). Auxin Depletion from Leaf Primordia Contributes to Organ Patterning. Proc. Natl. Acad. Sci. USA.

[33] Heisler M.G., Byrne M.E. (2020). Progress in Understanding the Role of Auxin in Lateral Organ Development in Plants. Curr. Opin. Plant Biol..

[34] Zhao F., Du F., Oliveri H., Zhou L., Ali O., Chen W., Feng S., Wang Q., Lü S., Long M. (2020). Microtubule-Mediated Wall Anisotropy Contributes to Leaf Blade Flattening. Curr. Biol..

[35] Silveira S.R., Le Gloanec C., Gómez-Felipe A., Routier-Kierzkowska A.-L., Kierzkowski D. (2022). Live-Imaging Provides an Atlas of Cellular Growth Dynamics in the Stamen. Plant Physiol..

[36] Sanders P.M., Bui A.Q., Weterings K., McIntire K.N., Hsu Y.-C., Lee P.Y., Truong M.T., Beals T.P., Goldberg R.B. (1999). Anther Developmental Defects in Arabidopsis thaliana Male-Sterile Mutants. Sex Plant Reprod..

[37] Ma H. (2005). Molecular Genetic Analyses of Microsporogenesis and Microgametogenesis in Flowering Plants. Annu. Rev. Plant Biol..

[38] Toriba T., Suzaki T., Yamaguchi T., Ohmori Y., Tsukaya H., Hirano H.-Y. (2010). Distinct Regulation of Adaxial-Abaxial Polarity in Anther Patterning in Rice. Plant Cell.

[39] Tian X., Li X., Yu Q., Zhao H., Song J., Liao J. (2020). Irregular Adaxial–Abaxial Polarity Rearrangement Contributes to the Monosymmetric-to-Asymmetric Transformation of Canna Indica Stamen. AoB Plants.

[40] Dinneny J.R., Weigel D., Yanofsky M.F. (2006). NUBBIN and JAGGED Define Stamen and Carpel Shape in Arabidopsis. Development.

[41] Sessions A., Nemhauser J.L., McColl A., Roe J.L., Feldmann K.A., Zambryski P.C. (1997). ETTIN Patterns the Arabidopsis Floral Meristem and Reproductive Organs. Development.

[42] Siegfried K.R., Eshed Y., Baum S.F., Otsuga D., Drews G.N., Bowman J.L. (1999). Members of the YABBY Gene Family Specify Abaxial Cell Fate in Arabidopsis. Development.

[43] Vandenbussche M., Horstman A., Zethof J., Koes R., Rijpkema A.S., Gerats T. (2009). Differential Recruitment of WOX Transcription Factors for Lateral Development and Organ Fusion in Petunia and Arabidopsis. Plant Cell.

[44] Nakata M., Matsumoto N., Tsugeki R., Rikirsch E., Laux T., Okada K. (2012). Roles of the Middle Domain–Specific WUSCHEL-RELATED HOMEOBOX Genes in Early Development of Leaves in Arabidopsis. Plant Cell.

[45] Vandenbussche M. (2021). The Role of WOX1 Genes in Blade Development and Beyond. J. Exp. Bot..

[46] Satterlee J.W., Evans L.J., Conlon B.R., Conklin P., Martinez-Gomez J., Yen J.R., Wu H., Sylvester A.W., Specht C.D., Cheng J. (2023). A Wox3-Patterning Module Organizes Planar Growth in Grass Leaves and Ligules. Nat. Plants.

[47] Matsumoto N., Okada K. (2001). A Homeobox Gene, PRESSED FLOWER, Regulates Lateral Axis-Dependent Development of Arabidopsis Flowers. Genes Dev..

[48] Åstrand J., Knight C., Robson J., Talle B., Wilson Z.A. (2021). Evolution and Diversity of the Angiosperm Anther: Trends in Function and Development. Plant Reprod..

[49] Hepworth S.R., Zhang Y., McKim S., Li X., Haughn G.W. (2005). BLADE-ON-PETIOLE-Dependent Signaling Controls Leaf and Floral Patterning in Arabidopsis. Plant Cell.

[50] Chen Q., Atkinson A., Otsuga D., Christensen T., Reynolds L., Drews G.N. (1999). The Arabidopsis FILAMENTOUS FLOWER Gene Is Required for Flower Formation. Development.

[51] Sawa S., Ito T., Shimura Y., Okada K. (1999). FILAMENTOUS FLOWER Controls the Formation and Development of Arabidopsis Inflorescences and Floral Meristems. Plant Cell.

[52] Eshed Y., Baum S.F., Perea J.V., Bowman J.L. (2001). Establishment of Polarity in Lateral Organs of Plants. Curr. Biol..

[53] Eshed Y., Izhaki A., Baum S.F., Floyd S.K., Bowman J.L. (2004). Asymmetric Leaf Development and Blade Expansion in Arabidopsis Are Mediated by KANADI and YABBY Activities. Development.

[54] Goldberg R.B., Beals T.P., Sanders P.M. (1993). Anther Development: Basic Principles and Practical Applications. Plant Cell.

[55] Walbot V., Egger R.L. (2016). Pre-Meiotic Anther Development: Cell Fate Specification and Differentiation. Annu. Rev. Plant Biol..

[56] Gould K.S., Lord E.M. (1988). Growth of Anthers in Lilium Longiflorum: A Kinematic Analysis. Planta.

[57] Kelliher T., Walbot V. (2011). Emergence and Patterning of the Five Cell Types of the *Zea Mays* Anther Locule. Dev. Biol..

[58] Jenik P.D., Irish V.F. (2000). Regulation of Cell Proliferation Patterns by Homeotic Genes during *Arabidopsis* Floral Development. Development.

[59] Lanfear R. (2018). Do Plants Have a Segregated Germline?. PLoS Biol..

[60] Burian A. (2021). Does Shoot Apical Meristem Function as the Germline in Safeguarding Against Excess of Mutations?. Front. Plant Sci..

[61] Marchant D.B., Walbot V. (2022). Anther Development—The Long Road to Making Pollen. Plant Cell.

[62] Xue J.-S., Yao C., Xu Q.-L., Sui C.-X., Jia X.-L., Hu W.-J., Lv Y.-L., Feng Y.-F., Peng Y.-J., Shen S.-Y. (2021). Development of the Middle Layer in the Anther of Arabidopsis. Front. Plant Sci..

[63] Van der Linde K., Walbot V. (2019). Pre-Meiotic Anther Development. Current Topics in Developmental Biology.

[64] Li Y., Ma H., Wu Y., Ma Y., Yang J., Li Y., Yue D., Zhang R., Kong J., Lindsey K. (2024). Single-Cell Transcriptome Atlas and Regulatory Dynamics in Developing Cotton Anthers. Adv. Sci..

[65] Nelms B., Walbot V. (2019). Defining the Developmental Program Leading to Meiosis in Maize. Science.

[66] Feng X., Dickinson H.G. (2007). Packaging the Male Germline in Plants. Trends Genet..

[67] Böwer F., Schnittger A. (2021). How to Switch from Mitosis to Meiosis: Regulation of Germline Entry in Plants. Annu. Rev. Genet..

[68] Cui D.-L., Xu C.-X., Wang P., Gao T.-Y., Wang B., Yu T.-Y. (2024). Male Gametogenesis in Flowering Plants. Front. Sustain. Food Syst..

[69] Schiefthaler U., Balasubramanian S., Sieber P., Chevalier D., Wisman E., Schneitz K. (1999). Molecular Analysis of NOZZLE, a Gene Involved in Pattern Formation and Early Sporogenesis during Sex Organ Development in *Arabidopsis thaliana*. Proc. Natl. Acad. Sci. USA.

[70] Yang W.C., Ye D., Xu J., Sundaresan V. (1999). The SPOROCYTELESS Gene of Arabidopsis Is Required for Initiation of Sporogenesis and Encodes a Novel Nuclear Protein. Genes Dev..

[71] Hao S., Ariizumi T., Ezura H. (2017). SEXUAL STERILITY Is Essential for Both Male and Female Gametogenesis in Tomato. Plant Cell Physiol..

[72] Rojas-Gracia P., Roque E., Medina M., Rochina M., Hamza R., Angarita-Díaz M.P., Moreno V., Pérez-Martín F., Lozano R., Cañas L. (2017). The Parthenocarpic Hydra Mutant Reveals a New Function for a SPOROCYTELESS-like Gene in the Control of Fruit Set in Tomato. New Phytol..

[73] Liu X., Ning K., Che G., Yan S., Han L., Gu R., Li Z., Weng Y., Zhang X. (2018). CsSPL Functions as an Adaptor between HD-ZIP III and CsWUS Transcription Factors Regulating Anther and Ovule Development in Cucumis Sativus (Cucumber). Plant J..

[74] Ren L., Tang D., Zhao T., Zhang F., Liu C., Xue Z., Shi W., Du G., Shen Y., Li Y. (2018). *OsSPL* Regulates Meiotic Fate Acquisition in Rice. New Phytol..

[75] Cui Y., Hu C., Zhu Y., Cheng K., Li X., Wei Z., Xue L., Lin F., Shi H., Yi J. (2018). CIK Receptor Kinases Determine Cell Fate Specification during Early Anther Development in Arabidopsis. Plant Cell.

[76] Cui Y., Lu X., Gou X. (2021). Receptor-like Protein Kinases in Plant Reproduction: Current Understanding and Future Perspectives. Plant Commun..

[77] Hu C., Zhu Y., Cui Y., Cheng K., Liang W., Wei Z., Zhu M., Yin H., Zeng L., Xiao Y. (2018). A Group of Receptor Kinases Are Essential for CLAVATA Signalling to Maintain Stem Cell Homeostasis. Nat. Plants.

[78] Soltabayeva A., Dauletova N., Serik S., Sandybek M., Omondi J.O., Kurmanbayeva A., Srivastava S. (2022). Receptor-like Kinases (LRR-RLKs) in Response of Plants to Biotic and Abiotic Stresses. Plants.

[79] Liu J., Li W., Wu G., Ali K. (2024). An Update on Evolutionary, Structural, and Functional Studies of Receptor-like Kinases in Plants. Front. Plant Sci..

[80] Lee H., Jun Y.S., Cha O.-K., Sheen J. (2019). Mitogen-Activated Protein Kinases MPK3 and MPK6 Are Required for Stem Cell Maintenance in the Arabidopsis Shoot Apical Meristem. Plant Cell Rep..

[81] Zhao F., Zheng Y.-F., Zeng T., Sun R., Yang J.-Y., Li Y., Ren D.-T., Ma H., Xu Z.-H., Bai S.-N. (2017). Phosphorylation of SPOROCYTELESS/NOZZLE by the MPK3/6 Kinase Is Required for Anther Development. Plant Physiol..

[82] Mohyeldin A., Garzón-Muvdi T., Quiñones-Hinojosa A. (2010). Oxygen in Stem Cell Biology: A Critical Component of the Stem Cell Niche. Cell Stem. Cell.

[83] Weits D.A., Kunkowska A.B., Kamps N.C.W., Portz K.M.S., Packbier N.K., Nemec Venza Z., Gaillochet C., Lohmann J.U., Pedersen O., Van Dongen J.T. (2019). An Apical Hypoxic Niche Sets the Pace of Shoot Meristem Activity. Nature.

[84] Kelliher T., Walbot V. (2012). Hypoxia Triggers Meiotic Fate Acquisition in Maize. Science.

[85] Xing S., Zachgo S. (2008). ROXY1 and ROXY2, Two Arabidopsis Glutaredoxin Genes, Are Required for Anther Development. Plant J..

[86] Hong L., Tang D., Zhu K., Wang K., Li M., Cheng Z. (2012). Somatic and Reproductive Cell Development in Rice Anther Is Regulated by a Putative Glutaredoxin. Plant Cell.

[87] De Reuille P.B., Bohn-Courseau I., Ljung K., Morin H., Carraro N., Godin C., Traas J. (2006). Computer Simulations Reveal Properties of the Cell-Cell Signaling Network at the Shoot Apex in Arabidopsis. Proc. Natl. Acad. Sci. USA.

[88] Vernoux T., Brunoud G., Farcot E., Morin V., Van den Daele H., Legrand J., Oliva M., Das P., Larrieu A., Wells D. (2014). The Auxin Signalling Network Translates Dynamic Input into Robust Patterning at the Shoot Apex. Mol. Syst. Biol..

[89] Ma Y., Miotk A., Šutiković Z., Ermakova O., Wenzl C., Medzihradszky A., Gaillochet C., Forner J., Utan G., Brackmann K. (2019). WUSCHEL Acts as an Auxin Response Rheostat to Maintain Apical Stem Cells in Arabidopsis. Nat. Commun..

[90] Yadav S., Kumar H., Mahajan M., Sahu S.K., Singh S.K., Yadav R.K. (2023). Local Auxin Biosynthesis Promotes Shoot Patterning and Stem Cell Differentiation in Arabidopsis Shoot Apex. Development.

[91] Ye Q., Zhu W., Li L., Zhang S., Yin Y., Ma H., Wang X. (2010). Brassinosteroids Control Male Fertility by Regulating the Expression of Key Genes Involved in Arabidopsis Anther and Pollen Development. Proc. Natl. Acad. Sci. USA.

[92] Chen W., Lv M., Wang Y., Wang P.-A., Cui Y., Li M., Wang R., Gou X., Li J. (2019). BES1 Is Activated by EMS1-TPD1-SERK1/2-Mediated Signaling to Control Tapetum Development in *Arabidopsis thaliana*. Nat. Commun..

[93] Shi L., Li C., Lv G., Li X., Feng W., Bi Y., Wang W., Wang Y., Zhu L., Tang W. (2024). The Adaptor Protein ECAP, the Co-Repressor LEUNIG, and the Transcription Factor BEH3 Interact and Regulate Microsporocyte Generation in Arabidopsis. Plant Cell.

[94] Gendron J.M., Liu J.-S., Fan M., Bai M.-Y., Wenkel S., Springer P.S., Barton M.K., Wang Z.-Y. (2012). Brassinosteroids Regulate Organ Boundary Formation in the Shoot Apical Meristem of Arabidopsis. Proc. Natl. Acad. Sci. USA.

[95] Ikeda M., Mitsuda N., Ohme-Takagi M. (2009). Arabidopsis WUSCHEL Is a Bifunctional Transcription Factor That Acts as a Repressor in Stem Cell Regulation and as an Activator in Floral Patterning. Plant Cell.

[96] Lenhard M., Bohnert A., Jürgens G., Laux T. (2001). Termination of Stem Cell Maintenance in *Arabidopsis* Floral Meristems by Interactions between *WUSCHEL* and *AGAMOUS*. Cell.

[97] Lohmann J.U., Hong R.L., Hobe M., Busch M.A., Parcy F., Simon R., Weigel D. (2001). A Molecular Link between Stem Cell Regulation and Floral Patterning in Arabidopsis. Cell.

[98] Sun B., Xu Y., Ng K.-H., Ito T. (2009). A Timing Mechanism for Stem Cell Maintenance and Differentiation in the Arabidopsis Floral Meristem. Genes Dev..

[99] Sun B., Looi L.-S., Guo S., He Z., Gan E.-S., Huang J., Xu Y., Wee W.-Y., Ito T. (2014). Timing Mechanism Dependent on Cell Division Is Invoked by Polycomb Eviction in Plant Stem Cells. Science.

[100] Ito T., Wellmer F., Yu H., Das P., Ito N., Alves-Ferreira M., Riechmann J.L., Meyerowitz E.M. (2004). The Homeotic Protein AGAMOUS Controls Microsporogenesis by Regulation of SPOROCYTELESS. Nature.

[101] Ito T., Ng K.-H., Lim T.-S., Yu H., Meyerowitz E.M. (2007). The Homeotic Protein AGAMOUS Controls Late Stamen Development by Regulating a Jasmonate Biosynthetic Gene in Arabidopsis. Plant Cell.

[102] Sieber P., Petrascheck M., Barberis A., Schneitz K. (2004). Organ Polarity in Arabidopsis. NOZZLE Physically Interacts with Members of the YABBY Family. Plant Physiol..

[103] Yang Q., Wang J., Zhang S., Zhan Y., Shen J., Chang F. (2023). ARF3-Mediated Regulation of SPL in Early Anther Morphogenesis: Maintaining Precise Spatial Distribution and Expression Level. Int. J. Mol. Sci..

[104] Juliano C., Wessel G. (2010). Versatile Germline Genes. Science.

[105] Varley Á., Horkan H.R., McMahon E.T., Krasovec G., Frank U. (2023). Pluripotent, Germ Cell Competent Adult Stem Cells Underlie Cnidarian Regenerative Ability and Clonal Growth. Curr. Biol..

[106] Greb T., Lohmann J.U. (2016). Plant Stem Cells. Curr. Biol..

[107] Bradamante G., Nguyen V.H., Incarbone M., Meir Z., Bente H., Donà M., Lettner N., Scheid O.M., Gutzat R. (2023). Two ARGONAUTE proteins loaded with transposon-derived small RNAs are associated with the reproductive cell lineage in Arabidopsis. Plant Cell.

[108] Meyerowitz E.M. (2002). Plants Compared to Animals: The Broadest Comparative Study of Development. Science.

[109] Watson J.M., Platzer A., Kazda A., Akimcheva S., Valuchova S., Nizhynska V., Nordborg M., Riha K. (2016). Germline Replications and Somatic Mutation Accumulation Are Independent of Vegetative Life Span in Arabidopsis. Proc. Natl. Acad. Sci. USA.

[110] Cammarata J., Morales Farfan C., Scanlon M.J., Roeder A.H.K. (2022). Cytokinin–CLAVATA Cross-Talk Is an Ancient Mechanism Regulating Shoot Meristem Homeostasis in Land Plants. Proc. Natl. Acad. Sci. USA.

[111] Nemec-Venza Z., Madden C., Stewart A., Liu W., Novák O., Pěnčík A., Cuming A.C., Kamisugi Y., Harrison C.J. (2022). CLAVATA Modulates Auxin Homeostasis and Transport to Regulate Stem Cell Identity and Plant Shape in a Moss. New Phytol..

[112] John A., Smith E.S., Jones D.S., Soyars C.L., Nimchuk Z.L. (2023). A Network of CLAVATA Receptors Buffers Auxin-Dependent Meristem Maintenance. Nat. Plants.

[113] Hirakawa Y., Fujimoto T., Ishida S., Uchida N., Sawa S., Kiyosue T., Ishizaki K., Nishihama R., Kohchi T., Bowman J.L. (2020). Induction of Multichotomous Branching by CLAVATA Peptide in Marchantia Polymorpha. Curr. Biol..

[114] Kohchi T., Yamato K.T., Ishizaki K., Yamaoka S., Nishihama R. (2021). Development and Molecular Genetics of Marchantia Polymorpha. Annu. Rev. Plant Biol..

[115] Benfey P.N. (2002). Auxin Action: Slogging out of the Swamp. Curr. Biol..

[116] Niederreither K., Dollé P. (2008). Retinoic Acid in Development: Towards an Integrated View. Nat. Rev. Genet..

[117] Koubova J., Menke D.B., Zhou Q., Capel B., Griswold M.D., Page D.C. (2006). Retinoic Acid Regulates Sex-Specific Timing of Meiotic Initiation in Mice. Proc. Natl. Acad. Sci. USA.

[118] Adolfi M.C., Herpin A., Regensburger M., Sacquegno J., Waxman J.S., Schartl M. (2016). Retinoic Acid and Meiosis Induction in Adult versus Embryonic Gonads of Medaka. Sci. Rep..

[119] Teletin M., Vernet N., Ghyselinck N.B., Mark M., Forrest D., Tsai S. (2017). Chapter Seven—Roles of Retinoic Acid in Germ Cell Differentiation. Current Topics in Developmental Biology.

[120] Walker C.H., Bennett T. (2019). A Distributive “50% Rule” Determines Floral Initiation Rates in the Brassicaceae. Nat. Plants.

[121] Coen E., Prusinkiewicz P. (2024). Developmental Timing in Plants. Nat. Commun..

[122] Li F., Yang J.-J., Sun Z.-Y., Wang L., Qi L.-Y., A S., Liu Y.-Q., Zhang H.-M., Dang L.-F., Wang S.-J. (2023). Plant-on-Chip: Core Morphogenesis Processes in the Tiny Plant Wolffia Australiana. PNAS Nexus.

